# Misidentification of Medical Devices With Radiographic Contrast Functions As Retained Foreign Bodies on Postoperative Radiographs: A Report of Two Cases

**DOI:** 10.7759/cureus.78154

**Published:** 2025-01-28

**Authors:** Shuji Uchimura, Toyoaki Maruta, Rintarou Kamada, Akiko Tomita, Isao Tsuneyoshi

**Affiliations:** 1 Anesthesiology, University of Miyazaki Hospital, Miyazaki, JPN; 2 Anesthesiology, Miyakonojo Medical Center, Miyakonojo, JPN; 3 Nursing, Miyakonojo Medical Center, Miyakonojo, JPN

**Keywords:** diagnostic error, human error, misidentification, patient safety, postoperative radiography, retained foreign body

## Abstract

Retained foreign bodies (RFBs) during surgery are events that should be completely avoided. Herein, we report two cases where a medical device with a radiographic contrast function was mistakenly identified as RFB due to human error. Radiographs were taken for confirmation to prevent foreign body retention after surgery. Case 1 involved a 71-year-old woman who underwent a laparoscopic bilateral adnexectomy for a right ovarian tumor. Postoperative abdominal radiography revealed a 5-mm spindle-shaped shadow in the pelvic cavity. Retention of a surgical instrument was suspected; however, no abnormalities were detected in the instruments used during surgery. Based on the foreign body's location, the shadow was assumed to be a bladder catheter. After removing the catheter, a repeat radiograph was performed, and the shadow disappeared. This bladder catheter had an X-ray contrast function and was misidentified as RFB. Case 2 involved a 38-year-old woman who underwent laparoscopic resection for pedicle torsion of a right ovarian tumor. Postoperative abdominal radiography revealed a contrast thread in the abdomen. A retained surgical gauze sponge was suspected; however, the instrument count was correct. The position of the gauze sponge was checked using mobile digital radiography equipment, and the object was identified as a cotton ball with an X-ray contrast thread placed in the umbilical wound. Additionally, the appearance of the cotton ball on the radiograph was unknown, which contributed to its misidentification as RFB. Eliminating human errors, including lack of communication and unfamiliarity with medical devices, is essential to prevent the misidentification of RFBs on postoperative radiographs.

## Introduction

Retained foreign body (RFB) after surgery is one of the most critical “never events” that should be completely avoided in surgical practice [1]. Despite implementing various measures to prevent RFB occurrences, they continue to be reported [2]. Complications such as intestinal perforation, sepsis, and death have been documented when RFBs occur after abdominal surgery. In some cases, reoperation is necessary to remove the RFB [3]. This results in prolonged hospitalization and increased patient suffering, which, in turn, undermines trust in medical care.

Although counting protocols and postoperative radiographs are standard practices at most institutions to prevent RFBs, such incidents continue to occur. Previous studies have shown that RFBs can occur even when counting is correct, highlighting the potential unreliability of manual counting [4]. A postoperative radiographic confirmation has proven effective in preventing RFBs [5], with radiopaque medical materials enhancing visibility. Rapid technological advancements over the past several decades have introduced numerous medical devices and materials. However, recognizing and interpreting these devices on radiographs has become increasingly challenging. Additionally, insufficient information about these materials can further complicate image interpretation [6].

We report two cases of mistaken RFB identification due to the unintentional use of radiopaque medical materials. These cases resulted from human error, as identifying these structures on radiographs proved difficult.

## Case presentation

Case 1

**Figure 1:**
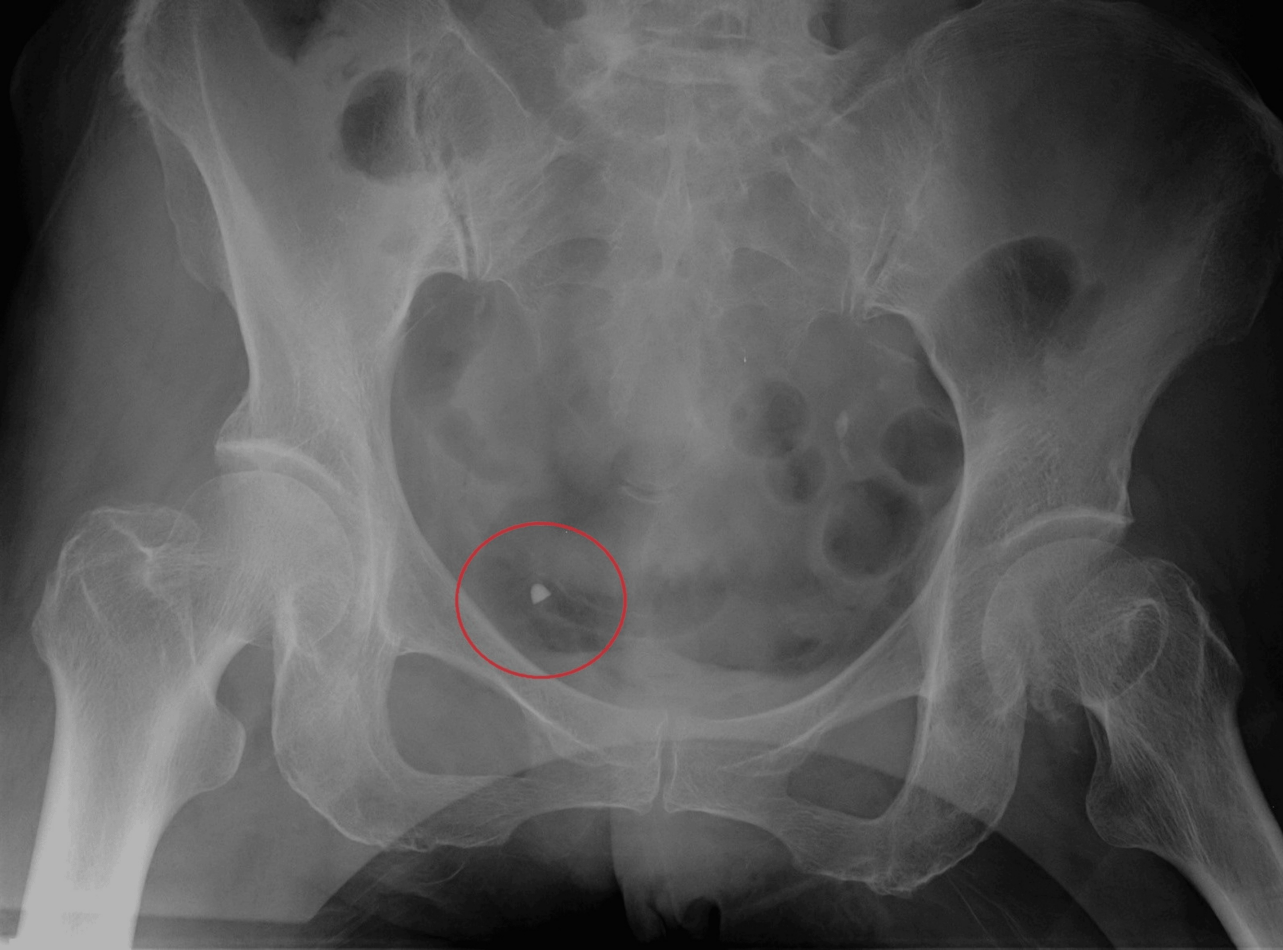
Postoperative abdominal radiograph (Case 1) Postoperative abdominal radiograph shows a 5 mm-sized spindle-shaped shadow in the pelvic cavity.

A 71-year-old woman underwent an emergency laparoscopic bilateral adnexectomy for a right ovarian tumor. The operation lasted 101 minutes and was completed as scheduled without any complications. Postoperative abdominal radiography to prevent RFBs revealed a 5-mm spindle-shaped shadow in the pelvic cavity. Suspecting residual surgical instruments, we rechecked the instruments used during the surgery; however, no abnormalities, such as missing or broken components, were found. Based on the location of the foreign body, we suspected the radiopaque tip of the indwelling bladder catheter. After removing the bladder catheter, a follow-up abdominal radiograph was obtained, and the shadow had disappeared. The bladder catheter (Create Medic Co., Ltd., All Silicone Foley Balloon Catheter 14Fr) used in this operation had been recently adopted, and the entire staff was unaware of its radiopaque tip, which led to its misidentification as an RFB (Figure 1).

Case 2

**Figure 2:**
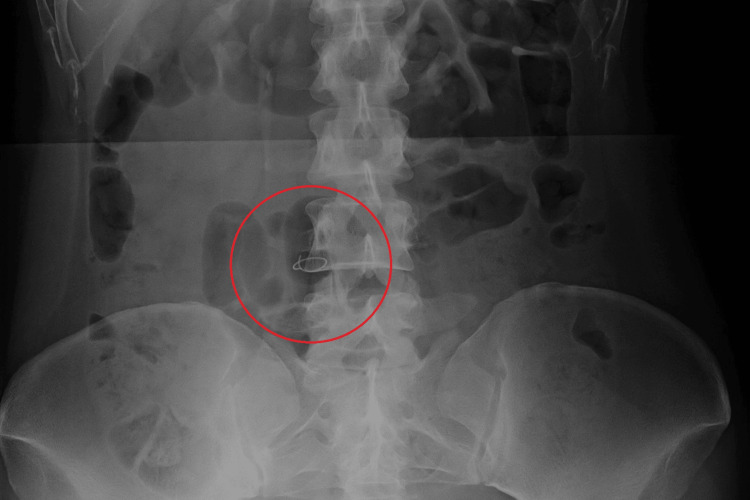
Postoperative abdominal radiograph (Case 2) Postoperative abdominal radiograph shows an 8 × 18 mm-sized X-ray contrast thread in the abdomen.

A 38-year-old woman underwent emergency laparoscopic ovarian tumor resection for right-sided ovarian torsion. The operation lasted 69 minutes and was completed as scheduled without any complications. Postoperative abdominal radiography revealed a radiopaque thread in the abdomen. Suspecting that surgical gauze had been left behind, the gauze count was repeated and found to be correct. After confirming the positional relationship using a mobile X-ray machine, it was suspected that a cotton ball with a radiopaque thread had been placed in the wound (umbilicus) on the body surface. Upon removal of the cotton ball, repeat abdominal radiography showed that the radiopaque thread had disappeared. This cotton ball is not typically used in gynecological procedures at our hospital; however, staff members mistakenly prepared it. The first scrub nurse noticed this error but failed to inform the next scrub nurse. Additionally, none of the staff knew how the cotton ball with the radiopaque thread would appear on radiographs. These factors led to its misidentification as an RFB (Figure 2).

## Discussion

“Never events” in surgery include operating on the wrong body part, performing surgery on the wrong patient, RFB in the patient’s body, and intraoperative or immediate postoperative death in patients classified as American Society of Anesthesiologists physical status I [1]. The incidence of RFBs is approximately one in 5,000 surgeries [7,8]. The risk of RFB significantly increases in cases of emergency surgery, unexpected changes in surgical procedures, and higher body mass index [3]. RFB occurrences are potentially life-threatening, and reoperations to remove RFBs are associated with a high mortality rate of 11-35% [9].

Interventions to prevent RFB occurrences include surgical counts, mandatory imaging after procedures, barcoding items used during surgery, and radiofrequency detection systems [10]. Manual counting has remained the mainstay for preventing hard RFBs, while intraoperative radiography is the most frequently used technology for identifying lost surgical sharps [4]. However, RFBs continue to be reported, including instances where RFBs occur even when counts are correct [5,7]. Postoperative radiography is an effective measure for preventing RFBs [5]. The use of radiopaque medical materials enhances visibility during imaging.

The rapid advancement of technology has led to the development of several new medical devices and materials. However, recognizing and interpreting these devices on radiographs has become increasingly challenging. Inadequate information about these materials often complicates their identification. We encountered two cases in which medical materials were mistakenly identified as RFBs due to the unintentional use of radiopaque medical materials caused by human error, making identifying these materials difficult.

Approximately 43% of operating room errors are attributed to miscommunication [11]. Miscommunication includes “occasion failures” (problems related to the situation or context of the communication event), “content failures” (insufficiencies or inaccuracies in the information being transferred), “audience failures” (gaps in the composition of the group engaged in the communication), and “purpose failures” (communication events in which the purpose is unclear, not achieved, or inappropriate) [12].

Case 1 involved an emergency surgery where the staff member who prepared the medical supplies differed from the staff member assigned as the scrub nurse. The staff member who managed the supplies was absent, which led to the radiopaque tip of the indwelling bladder catheter being mistaken for RFB. Following this incident, all staff members were reminded about medical devices with radiographic contrast functions used in the operating room, and workshops were conducted to familiarize them with the new medical devices. Some have asserted that the imaging appearances of surgical materials in the postoperative abdomen and pelvis can be confusing and difficult to interpret. Radiologists must be familiar with the imaging characteristics of various intentionally and unintentionally placed surgical materials and devices [13]. This familiarity is also important for anesthesiologists, who often have the opportunity to evaluate postoperative radiographs. Anesthesiologists should be well-acquainted with the radiographic appearances of devices routinely used in the operating room.

Case 2 was another emergency surgery where, as in Case 1, the staff member who prepared the medical supplies differed from the scrub nurse. The first scrub nurse failed to inform the next scrub nurse that an incorrect cotton ball had been prepared. Consequently, this incident was caused by a communication error. Effective teamwork and communication among healthcare providers are critical for ensuring quality and safe patient care. To achieve desired outcomes, teamwork requires cooperation, coordination, and communication among team members [14].

After this incident, a protocol was established whereby any unscheduled events were immediately communicated to all surgical team members, even during surgery. Additionally, since communication errors are more likely to occur during emergency surgeries, all staff members were reminded to communicate more frequently in such situations.

## Conclusions

Owing to human error, we encountered two cases in which medical devices with radiographic contrast functions were unintentionally used and mistakenly identified as RFBs, as it was difficult to recognize the medical devices. When reviewing postoperative radiographs, it is essential to acquire knowledge of the equipment used and to eliminate communication errors among staff members regarding the medical materials used during the procedure. However, no safety measures are entirely foolproof, and all staff must remain acutely aware of the RFB problem to prevent its occurrence. Since our report is based on only two cases, further studies are warranted.
